# Recent Developments in Peptide-Based Nucleic Acid Delivery

**DOI:** 10.3390/ijms9071276

**Published:** 2008-07-16

**Authors:** Sandra Veldhoen, Sandra D. Laufer, Tobias Restle

**Affiliations:** 1 Department of Metabolomics, ISAS - Institute for Analytical Sciences, Bunsen-Kirchhoff-Str. 11, 44139 Dortmund, Germany; 2 Institut für Molekulare Medizin, Universitätsklinikum Schleswig-Holstein, Universität zu Lübeck, Ratzeburger Allee 160, 23538 Lübeck, Germany

**Keywords:** cell-penetrating peptides, nucleic acid drugs, nucleic acid delivery, endocytosis, bPrPp, bovine prion protein derived peptide, CLSM, confocal laser scanning microscopy, CPP, cell-penetrating peptide, FCS, fetal calf serum, EIPA, ethylisopropylamiloride, GFP, green fluorescent protein, hCT, human calcitonin, HEPES, 4-(2-hydroxyethyl)-1-piperazineethanesulfonic acid, HIV, human immunodeficiency virus, IL, interleukin, IFN, interferon, LF, Lipofectamine™, LF2000, Lipofectamine™ 2000, MAP, model amphipathic peptide, MEND, multifunctional envelope-type nano device, miRNA, microRNA, mPrPp, murine prion protein derived peptide, NLS, nuclear localisation sequence, OMe, O-methyl, PAMAM, polyamidoamine, PEG, polyethylene glycol, PEI, polyethyleneimine, PMO, phosphorodiamidate morpholino oligomer, PNA, peptide nucleic acid, PTD, protein transduction domains, RNAi, RNA interference, SAP, Sweet Arrow Peptide, siRNA, small inhibitory RNA, STR-R_8_, stearyl-R_8_, TAR, transactivator responsive region, TFO, triplex forming oligonucleotide, TLR9, toll-like receptor 9, TNF, tumour necrosis factor, TP10, transportan 10

## Abstract

Despite the fact that non-viral nucleic acid delivery systems are generally considered to be less efficient than viral vectors, they have gained much interest in recent years due to their superior safety profile compared to their viral counterpart. Among these synthetic vectors are cationic polymers, branched dendrimers, cationic liposomes and cell-penetrating peptides (CPPs). The latter represent an assortment of fairly unrelated sequences essentially characterised by a high content of basic amino acids and a length of 10–30 residues. CPPs are capable of mediating the cellular uptake of hydrophilic macromolecules like peptides and nucleic acids (e.g. siRNAs, aptamers and antisense-oligonucleotides), which are internalised by cells at a very low rate when applied alone. Up to now, numerous sequences have been reported to show cell-penetrating properties and many of them have been used to successfully transport a variety of different cargos into mammalian cells. In recent years, it has become apparent that endocytosis is a major route of internalisation even though the mechanisms underlying the cellular translocation of CPPs are poorly understood and still subject to controversial discussions. In this review, we will summarise the latest developments in peptide-based cellular delivery of nucleic acid cargos. We will discuss different mechanisms of entry, the intracellular fate of the cargo, correlation studies of uptake *versus* biological activity of the cargo as well as technical problems and pitfalls.

## 1. Introduction

**Figure 1.**
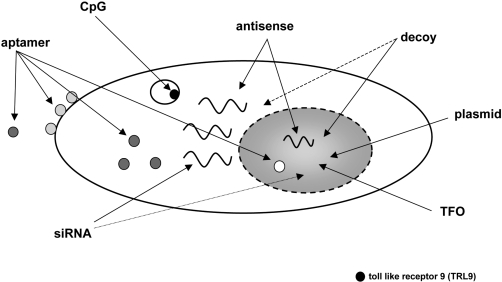
**Cellular target sites of oligonucleotides with therapeutic potential.** Aptamers, small oligonucleotides derived from an *in vitro* evolution process called SELEX, can virtually be targeted to any given extra- or intracellular structure. Oligonucleotides containing a CpG motif interact with toll-like receptor 9 (TLR9) and trigger an immunostimulatory response. Antisense and decoy oligonucleotides as well as siRNAs can modulate gene expression by interacting with RNA or proteins either in the cytoplasm or in the nucleus. TFOs are directed against genomic DNA and, like plasmids, have to reach the nucleus to exert their biological effect.

Today there is a fast growing number of nucleic acid-based strategies to modulate a vast variety of cellular functions (for a review see: [1,2]). Several classes of oligonucleotides like aptamers, transcription factor-binding decoy oligonucleotides, ribozymes, triplex-forming oligonucleotides (TFO), immunostimulatory CpG motifs, antisense oligonucleotides including peptide nucleic acids (PNAs), small interfering RNAs (siRNAs) and antagomirs have attained much interest as a research tool owing to their highly specific mode of action. Even more important, these oligomeric nucleic acids do have a considerable potential to be used as therapeutics. Figure 1 provides an overview of such oligonucleotides and their target sites within the cell.

Although quite different in their mode of action, oligomeric nucleic acids have several features in common. Essentially, they can either be rationally designed (e.g. antisense oligonucleotides or siRNAs) or selected *in vitro* (e.g. aptamers or ribozymes). These are major advantages compared to traditional small molecule drug screening approaches. In general, these macromolecules show remarkably high specificity for their targets accompanied by low probability of generating side-effects. Additionally, nucleic acids are virtually non-immunogenic compared to protein- or peptide-based approaches. On the downside, considerations like stability, bio-availability and pharmacokinetics come into play. Though, these drawbacks can be resolved by appropriate chemical modifications. Nuclease resistance for instance can be achieved by alkyl modifications at the 2′-position of the ribose. In recent years, valuable progress has been accomplished through the development of novel chemically modified nucleotides with improved properties such as enhanced serum stability, higher target affinity and low toxicity. Pharmacokinetic parameters can be rationally improved by increasing the molecular size, e.g. by site-specific conjugation of polyethylene glycol (PEG). In spite of this, the major obstacle for turning oligomeric nucleic acids into drugs is efficient cellular delivery. Due to this limitation, for a long time nucleic acids were not considered to have a significant therapeutic prospective, though their efficiency has been proven by countless *in vitro* studies. This obvious dilemma urgently calls for safe and efficient nucleic acid delivery systems.

**Figure 2.**
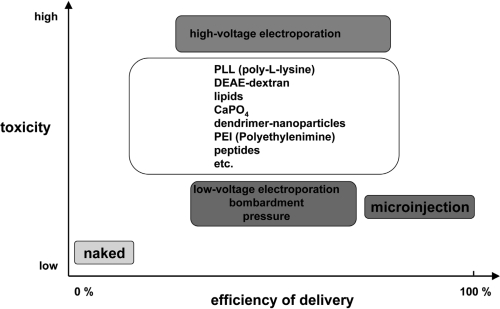
Comparison of delivery efficiency *versus* toxicity for various DNA transfection methods. Figure adapted from  [9].

Essentially, the nucleic acid delivery techniques available today comprise various physical and chemical methods, viral and non-viral vector systems, and uptake of naked nucleic acids (Figure 2). They all have certain advantages and disadvantages and might only be appropriate if particular requirements are fulfilled. In general, physical and chemical methods like microinjection, electroporation or particle bombardment as well as calcium phosphate coprecipitation are highly efficient but rather harmful for the target cells and lack the potential to be applicable *in vivo*. There is general consent that viral vector systems are the most efficient vehicles to deliver nucleic acids into cells. However, despite substantial efforts over the last 15 years, up to now research has failed to develop suitable and especially safe viral systems (for a review see: [3,4]). On the contrary, the field has experienced several setbacks causing important clinical trials to be put on hold [5–8]. As a result of the difficulties encountered with these viral vectors (e.g. mutagenesis and immune responses) much attention was paid to the development of allegedly safer non-viral delivery systems. This conception includes an assortment of fairly unrelated approaches yielding various degrees of enhanced cellular uptake of nucleic acids. Currently, liposomes and cationic polymers are used as a standard tool to transfect cells *in vitro*. These approaches are yet characterised by a significant lack of efficiency accompanied by a high level of toxicity rendering them mostly inadequate for *in vivo* applications.

Peptides acting as shuttles for a controlled cellular delivery of nucleic acids represent a new and innovative concept to bypass the problem of poor bio-availability and clinical efficacy of such macromolecules. The idea of using peptides as carriers goes back some 20 years when two groups discovered by chance that the HIV-1 transactivating protein Tat is taken up by mammalian cells [10,11]. Just a few years later, the Antennapedia homeodomain of *Drosophila melanogaster* was shown to act similarly [12]. Later on, it could be shown that peptides derived from Tat and Antennapedia as well as other proteins are capable of transporting macromolecular cargo molecules into cells [13–15]. Based on such promising results, a rapidly expanding field focusing on the so-called cell-penetrating peptides (CPPs), also referred to as protein transduction domains (PTD) began to develop.

In this review we will report about recent progress in the field of peptide-mediated delivery of nucleic acids, highlighting the development of several new CPPs, and discuss mechanisms for cellular internalisation. Additionally, we will present own data on peptide-mediated siRNA delivery and briefly discuss them in the given context.

## 2. General Properties of CPPs

Up to now numerous CPPs have been described. According to their origin, they can be grouped into three classes. The first group comprises CPPs originating from naturally occurring proteins (‘protein derived CPPs’), the second consists of ‘chimeric CPPs’ composed of different protein domains and the third class encompasses so-called ‘model CPPs’ which were developed according to structure-function relationships without any homology to natural sequences. Common to all known CPPs are basic amino acids causing a net positive charge at physiological pH. In a first attempt to define a peptide as a CPP the following definition was established [16]: (I) CPPs consist of less than 30 amino acids; (II) CPPs are internalised by cells in a seemingly receptor- and protein-independent process, even at 4 °C; (III) CPPs are able to mediate the delivery of a cargo. As will be discussed later on, this definition is no longer appropriate. At present, a peptide is considered a CPP, if it shows the ability to ‘cross’ a biological membrane. A cargo can be bound to the CPP covalently or non-covalently. Covalent attachment can be achieved either by expression as a fusion construct or by chemical coupling (for a review see: [17]). In some cases, cargo and carrier bind each other non-covalently through ionic interactions. Depending on the nature of both binding partners assembly of nanoparticles may occur.

## 3. Mechanism of cellular translocation

**Figure 3.**
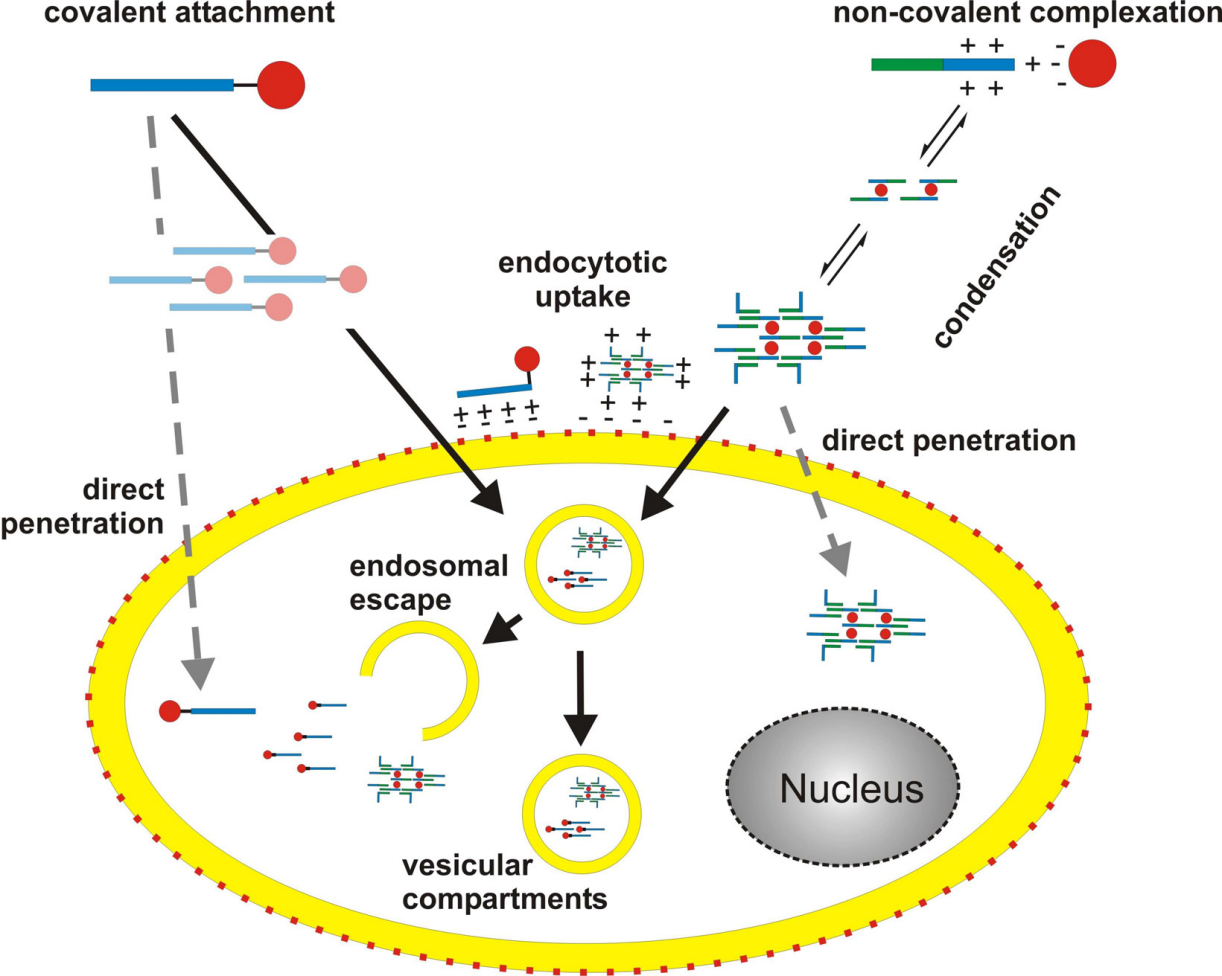
**Principles of peptide-based nucleic acid delivery systems.** Interaction of CPP and cargo is either achieved by covalent attachment or by non-covalent complexation through mainly ionic interactions. In case of non-covalent complex formation, a further assembly of cargo/carrier complexes occurs, leading to the formation of nanoparticles. In case of covalently joined molecules a similar scenario is less likely, yet cannot be excluded. Prior to the translocation process the particles attach to the cell surface by ionic interactions of positively charged CPP residues with negatively charged membrane components. Subsequently, complexes are taken up by directly penetrating the cell membrane or by an endocytotic pathway. Recent data suggest that the main uptake route is endocytosis. Though, direct penetration cannot be excluded and may occur simultaneously (depicted by dashed, grey arrows). Once inside the cell, the cargo has to reach its target. Depending on the mechanism of uptake several scenarios like ‘endosomal escape’ are feasible. Red: negative charges, blue: positive charges, green: hydrophobic domains.

Despite the widespread interest in peptide carriers the mechanisms underlying the cellular translocation of CPPs are poorly understood and subject to controversial discussions (Figure 3). Early work relied upon fluorescence imaging or flow cytometry analysis of chemically fixed cells to examine intracellular localisation of fluorescently labelled peptides in the absence or presence of cargo. According to these experiments peptides seemed to be internalised very rapidly within minutes even at 4 °C. From such observations it was concluded that CPPs penetrate cell membranes by an energy-independent mechanism [18–22]. Although it had been reported quite early on that certain fixation procedures may cause artefacts leading to an overestimation of the cellular uptake rates [23–25] the dimension of this problem was not commonly recognised until a side by side comparison of fixed and living cells was published [26]. The authors convincingly demonstrated that the distribution pattern of Tat^48–60^ and R_9_ as well as their conjugates was completely different in living *versus* fixed cells. They stress that an important source of misinterpretation arises from experimental difficulties to distinguish cell surface-associated CPP from CPP internalised into cytoplasmic compartments. Most notably it could be shown that endocytotic transport is a key component of the internalisation process of a Tat derived peptide [26] and penetratin [27]. Prior to endocytosis CPPs interact electrostatically with the extracellular matrix of the cell surface mostly through binding to negatively charged glycosaminoglycans, i.e. heparan sulfate proteoglycans [28–31].

**Figure 4.**
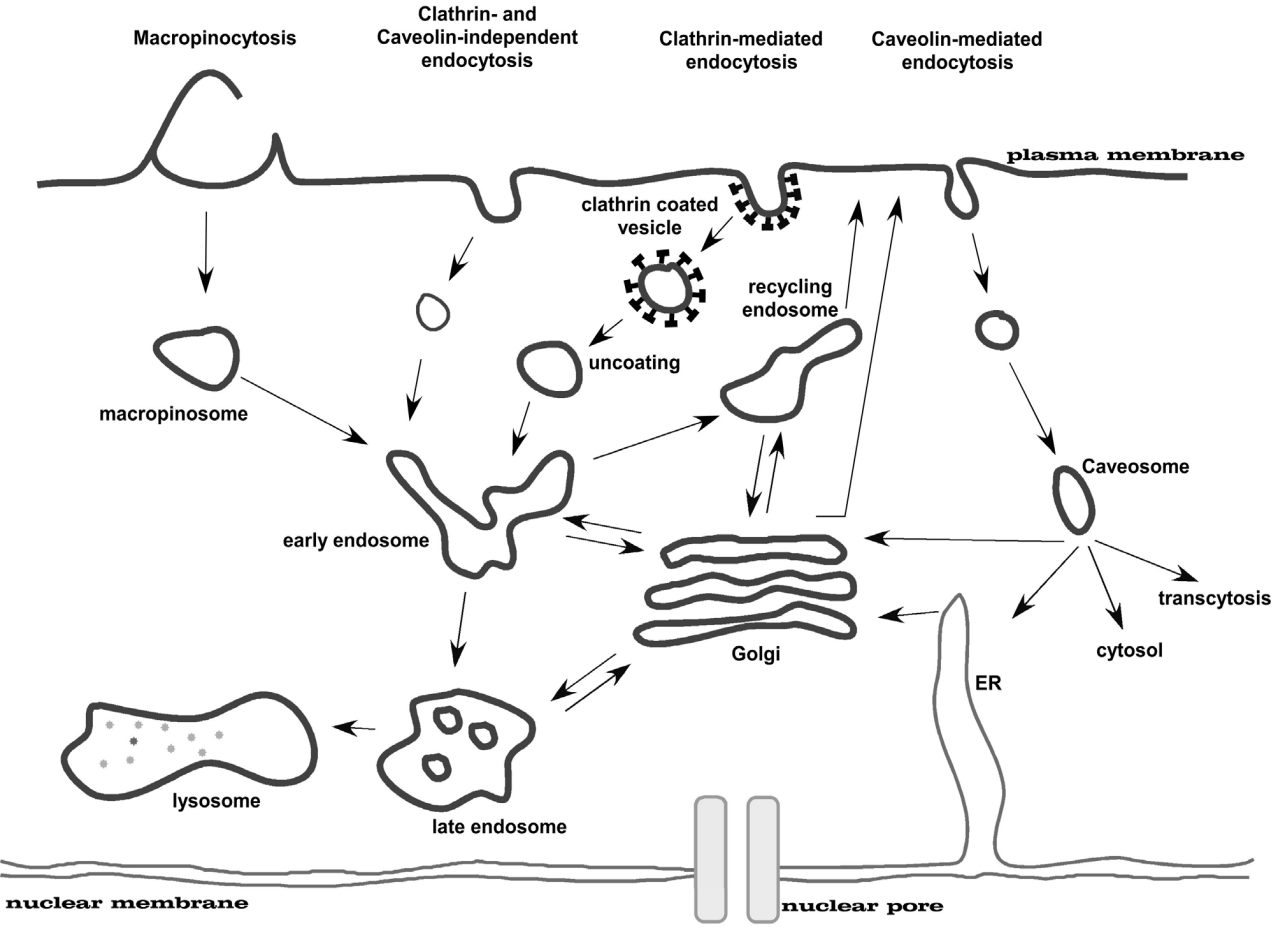
**Multiple portals of entry into the mammalian cell.** The endocytic pathways differ with regard to the size of the endocytic vesicle, the nature of the cargo (ligands, receptors and lipids) and the mechanism of vesicle formation. Figure adapted from [35].

Eukaryotic cells take up macromolecules and particles from the surrounding medium by a process called endocytosis. Besides phagocytosis, which is only relevant for specialised cells like macrophages, this process occurs in all cells by at least four basic mechanisms: macropinocytosis, clathrin-mediated endocytosis, caveolin-mediated endocytosis, and clathrin- and caveolin-independent endocytosis (Figure 4; for a review see: [32,33]). These mechanistically diverse and highly regulated endocytic pathways contribute to control complex physiological processes, such as hormone-mediated signal transduction, immune surveillance, antigen-presentation, and homeostasis. The best-studied endocytosis pathway is the clathrin-coated pit pathway (for a review see: [34]). Many receptors and their associated ligands cluster into clathrin-coated pits by association with clathrin adaptor proteins such as the four-subunit complex AP2. Clathrin adaptors in turn bind to the clathrin lattice which is thought to provide the force required to deform the membrane into a curved bud. The large GTPase dynamin is then involved in pinching off the coated pit to form a clathrin-coated vesicle. Such vesicles are then uncoated by the chaperone hsc70 and the DNA-J domain co-chaperone auxillin. Uncoated endocytic vesicles then fuse with one another and with early endosomes in a reaction requiring the small GTPase Rab5. Eventually, early to late endosome transport may be mediated by small vesicular intermediates. Late endosomes are then thought to fuse with pre-lysosomes to form ‘hybrid’ organelles which mature back into lysosomes through sorting and fission.

The second best studied pathway depends on caveolin (for a review see: [36]). Oligomerised caveolin associates at the plasma membrane with proteins, e.g. PTRF-cavin [37], and lipids to form stable functional units referred to as caveolae. This caveolar unit is also maintained as a stable structure upon endocytosis. Budded caveolae can fuse either with the caveosome in a Rab5-independent manner or with the early endosome in a Rab5-dependent manner. Caveolae remain at the plasma membrane for long periods, but various agents can stimulate their internalisation. These include the SV40 virus as well as sterols and glycosphingolipids which use caveolae for entry into cells and stimulate caveolar budding. Despite the different agents used to stimulate caveolar internalisation there are common mechanisms involved in these pathways with a crucial role for dynamin, Src kinases, protein kinase C and actin recruitment. Besides these two pathways, there are other less well-defined pathways (i.e. clathrin- and caveolin-independent, lipid-raft endocytosis) that differ from the routes described above [32].

As most of these pathways have several characteristics in common, it remains complicated to precisely determine which endosomal route is followed [38–40]. This in part can be attributed to the fact that endocytosis itself is far from being understood in detail. Differentiation between alternative endocytosis pathways involved in cellular uptake of cargo molecules can be accomplished by the use of small molecule inhibitors or by colocalisation studies with specific markers following fluorescence microscopy or cell fractionation. As the fate of the cargo (e.g. degradation in lysosomes, storage in caveosomes or transport to the Golgi complex or the ER etc.) largely depends on which endocytosis pathway is involved in uptake, it would be highly desirable to induce internalisation *via* a particular pathway in order to better control trafficking through the cell. Currently, this is not easily achieved. However, there are certain parameters which influence the mode of uptake like particle size, the interaction of the complexes with cell surface components as well as the physiological state of the target cells. In that respect there are several options to manipulate cell trafficking.

Based on the findings described above, many groups began to re-examine their data. However, despite considerable technical improvements, there are still puzzling controversial results concerning the exact mechanism of CPP uptake. Though in most cases endocytosis has been suggested to be the main route of internalisation, substantial difficulties are encountered identifying the exact pathway. Recent studies indicate that the uptake mechanism of CPPs can be influenced by the attachment of cargos. For example, Richard *et al*. [26,41] reported a co-localisation of Tat^48–59^ with markers of clathrin-mediated endocytosis, whereas Fittipaldi *et al*. [42] found a caveolae/lipid raft-dependent process for a Tat-GFP fusion protein and Wadia *et al*. [43] described a macropinocytotic uptake pathway for a fusion construct of Tat peptide with Cre recombinase.

To avoid the complexity inherent to cell culture-based experiments, biophysical investigations of CPP/membrane interactions with different model membranes were performed. According to these kind of studies, a direct penetration of several CPPs through the lipid layer was proposed [44–48]. However, it is highly problematic to transfer these data to the cellular level, as natural membranes are composed of a heterogeneous mixture of proteins and lipids including distinctive microdomains such as lipid rafts (microdomain enriched in cholesterol and sphingolipids [49]). What is even more important, cells are surrounded by an extracellular matrix consisting of miscellaneous proteins and receptors. Thus, it is obvious that simple model membranes do not reflect this situation. Nevertheless, such studies could in part mimic processes taking place at endosomal membranes and might help to understand how CPPs escape from these vesicles.

Taken together, it has to be concluded that the precise mechanism of internalisation remains elusive and strongly depends on the properties of both CPP and cargo as well as on the transfection conditions and the cell lines used [50–54]. Furthermore, it is reasonable to speculate about multiple pathways involved in cellular entry and that when blocking one of them an alternative pathway may become active [54].

## 4. Properties of selected CPPs

In the following section we will briefly describe main features of selected CPPs. In the first part examples of ‘classical’ CPPs are given. The second part highlights more recent developments in the field. There, a selection of several new peptide sequences and concepts will be presented. Additional information on the most important CPPs can be found in a number of reviews [55–59]. Lochmann *et al*. [60] give a list of studies concerning peptide-mediated delivery of oligonucleotides and a more comprehensive description of the cellular delivery of bioactive cargos is provided by Dietz *et al*. [61]. Järver *et al*. recently reported on the most recent applications of CPPs for the regulation of gene expression [62]. Table 1 and 2 give an overview of selected peptides.

### 4.1. Classical CPPs

#### HIV-1 Tat and derivatives

The ability of the HIV-1 transactivator protein (Tat) to penetrate cellular membranes was first discovered in 1988 [10,11]. Since then, numerous reports have described a successful intracellular transport of several macromolecular cargos, including entire proteins like β-galactosidase or horseradish peroxidase [13,15], covalently attached to this protein or its fragments (for a recent review see: [77–80]). *In vivo*, these cargo proteins were found in an enzymatically active form in cells of several organs including the brain. By testing different truncated versions of Tat, Vives *et al*. [20] revealed that Tat^48–60^ had the highest transfection efficiency. An even shorter peptide almost exclusively composed of basic amino acids (Tat^49–57^) proved to be essential and sufficient for nuclear import in mammalian cells. Here, the guanidinium group was shown to be superior to other cationic groups in the sequence [81]. Conjugates of Tat with proteins [13,15], peptides [82–84], PNA [85], various classes of oligonucleotides [86–89], and siRNA [90] have been shown to translocate into cells. Moreover, the transport of non-covalently bound plasmid DNA [91–93], liposomes [94–96], and even adenoviral particles [97] has been demonstrated.

#### Penetratin

Penetratin, formerly termed pAntp^43–58^, is a peptide derived from the third helix of the *Drosophila melanogaster* Antennapedia homeodomain protein [63,98]. It is one of the most widely investigated CPPs exerting low toxicity and a high translocation rate. As for Tat, its translocation efficiency strongly depends on certain basic residues. In spite of this, retro- and enantio-modified versions showed comparable properties in earlier studies [18,99], which has been interpreted as evidence for a receptor-independent uptake mechanism. In contrast to this, Báránye-Wallje *et al*. recently presented convincing data that penetratin is not able to directly traverse a number of lipid bilayer model membranes [100]. According to this study, cellular uptake of penetratin is supposed to occur *via* endocytic pathways probably initiated by electrostatic interactions with cell surface molecules. This is also in accordance with *in vitro* studies showing that the cellular internalisation process is temperature- as well as energy-dependent and can be influenced by various endocytosis inhibitors [27,31]. Meanwhile, there are numerous reports on the cellular delivery of covalently attached cargos like proteins or peptides [101–103] and oligonucleotides including PNAs and siRNAs [85,87,104–108]. Promising results were recently achieved with a modified version of penetratin concerning the endosomal release of internalised siRNA [76] and with the attachment of a hexaarginine (R6) to the N-terminus of penetratin concerning the delivery of a splice correcting PNA in the absence of endosomolytic agents [109].

#### Transportan and derivatives

Transportan, a chimeric 27 amino acid peptide, was originally developed by the group of Langel [64]. It is composed of the N-terminal amino acids of the neuropeptide galanin, coupled to mastoparan, a peptide from wasp venom which strongly interacts with membranes [64]. Trimming down transportan led to TP10, a peptide with 21 residues displaying similar properties as the initial peptide [65]. Transportan and some of its analogues were shown to deliver proteins up to 150 kDa [110], plasmids [111] and oligonucleotides, including siRNAs [104] and PNAs [112–114]. For the latter application it was shown in two independent comparative studies that transportan is even more potent in delivering a conjugated PNA for splice correction than penetratin or Tat [105,115]. To elucidate the mechanism of translocation, Padari *et al*. studied the cellular uptake of biotinylated transportan or TP10 complexed with labelled avidin conjugates by transmission electron microscopy and confocal laser scanning microscopy [116]. The internalisation process was temperature-dependent (i.e. strongly inhibited at 10 °C and blocked at 4 °C) and complexes were predominantly observed in endocytotic vesicles of different morphology and size.

#### Oligoarginines

Microscopic studies performed by Futaki *et al*. [22] revealed that oligoarginines and other arginine-rich peptides are efficiently taken up by cells. The molecule with the highest efficiency turned out to be octaarginine (R_8_), whereas peptides of < 5 and > 12 arginine residues showed only negligible translocation [117]. Studies with several branched versions of oligoarginine indicated that the number of arginine residues is much more important than the structure *per se* [118]. A direct comparison of nonamers composed of arginine, histidine, lysine or ornithine revealed that arginine residues were most effective in penetrating the plasma membrane because of their guanidinium group [66,81]. It was shown that an apoptosis-inducing peptide conjugated to R_8_ was active in HeLa cells after translocation [119]. Furthermore, modified oligoarginines were used for non-covalent delivery of plasmids [120] and siRNAs [121]. Nakase *et al*. [122,123] demonstrated a macropinocytosis-dependent internalisation pathway for R_8_. These data are based on the observation that ethylisopropylamiloride (EIPA), a macropinocytosis specific inhibitor, and the F-actin polymerisation inhibitor cytochalasin D significantly suppressed uptake. Additionally, activation of Rac protein and rearrangement of the actin cytoskeleton was observed within a few minutes after incubation with the peptide. Interestingly, flow cytometry analysis revealed that R_9_ was internalised to a similar degree as penetratin, but to a significantly larger extent than Tat peptide [124].

#### Model amphipathic peptides

Model amphipathic peptides (MAP, also termed KLA peptides) are derived from the α-helical amphipathic model peptide KLALKLALKALKAALKLA, designed by Steiner *et al*. [125]. This peptide has been shown to internalise by multiple, non-specific, energy-dependent and -independent processes into several types of cells [67]. Additionally, transport of covalently attached short peptides and PNAs as well as transport of oligonucleotides attached either covalently or non-covalently into mammalian cells was reported. [67,126,127]. Using a splice correcting PNA and different KLA analogues, Wolf *et al*. further analysed the influence of charge and structure-forming properties of the conjugate on the antisense effect [128]. Various constructs of unstructured, non-amphipathic or negatively charged peptides with the same PNA showed less or no activity at all. Moreover, replacing arginine residues by lysines did not improve antisense activity and a cleavable disulfide was not required for efficient splice correction, whereas a localisation of the peptide at the N-terminus of the PNA was crucial for attaining antisense activity. The predominant portion of PNA-KLA conjugates seems to be taken up by endocytosis, as coadministration of lysosomotropic agents [128] as well as photochemical treatment [129] promoted a significant enhancement of the observed antisense effects.

#### MPG and Pep families

MPG is a 27 amino acid peptide composed of a hydrophobic domain derived from the N-terminal fusion sequence of the HIV-1 glycoprotein 41 and a hydrophilic domain derived from the nuclear localisation sequence (NLS) of the SV40 large T-antigen which are linked by a 3 amino acid spacer [19]. Unlike most CPPs described today, this peptide does not need covalent attachment of the cargo molecule. Complex formation with the cargo occurs through ionic interactions of the positively charged NLS sequence and negative charges of the cargo. In addition, depending on the nature of the cargo, hydrophobic interactions may be important as well. Furthermore, hydrophobic peptide/peptide interactions lead to the formation of nanoparticles. Fluorescence measurements revealed apparent dissociation constants in the low nanomolar range for nucleic acid/peptide interactions with an approximately 20-fold excess of positive charges. MPG was shown to deliver DNA oligonucleotides, plasmid DNA, and siRNA into mammalian cells [19,130,131]. More recently, different derivatives of MPG have been described [68,132–135]. One peptide, termed MPGα differs by 6 amino acids in the hydrophobic part. As opposed to the original peptide, these modifications lead to a predominantly α-helical structure, whereas MPG is non-ordered in water and adopts a β-sheet-like structure in contact with oligonucleotides or phospholipids [19,68]. Both peptides show the ability to insert spontaneously into model membranes. This phenomenon has been proposed to be a prerequisite for their cell-penetrating activity. Further variations in the hydrophobic part of the original MPG sequence led to the Pep-family. These peptides were designed for the delivery of proteins, peptides [132] and PNA analogues [133,134]. For all peptides except MPGα a non-endosomal entry mechanism has been proposed [136,137].

### 4.2. New peptides and concepts

#### Human calcitonin and derivatives

Human calcitonin (hCT) is a 32 amino acid peptide hormone involved in the regulation of calcium homeostasis. It is therapeutically used in osteoporosis and other bone-related diseases (for a review see: [138]). Remarkably, direct application of hCT to the nasal epithelium has been proposed to be as effective as intravenous injections [139]. The investigation of several shorter versions of hCT revealed that the shortest fragment taken up by cells is hCT^9–32^, probably *via* endocytosis [140]. Attachment of the SV40 large T-antigen NLS to the side chain of Lys^18^ in hCT^9–32^ resulted in a branched peptide (hCT^9–32^-br) which showed the ability to promote internalisation of plasmids into human neuroblastoma cells by formation of complexes through non-covalent interactions [69]. The rate of transfection was significantly higher than the efficiency observed for the commercial transfection reagent Lipofectamine™ (LF) or a linear fusion of hCT^9–32^ to the SV40-NLS. The mechanism of uptake has not yet been resolved in detail, however, studies with labelled hCT^9–32^-br indicate lipid raft-mediated endocytosis [141]. As the performance of hCT^9–32^-br for many other cell lines was rather low, this concept was further optimised leading to several branched hCT-derived peptides [142]. The efficiency of transfection after non-covalent complexation with plasmids was highest for a peptide with a branch consisting of two SV40-derived NLS called hCT^9–32^-2br and for hCT^18–32^-k7 in which both parts were truncated to form a peptide with only 28 amino acids. Furthermore, cell line specific differences became obvious in this study as hCT^9–32^-2br showed the highest transfection rate for HEK 293 cells and rat hippocampal neurons whereas hCT^18–32^-k7 was more efficient for neuroblastoma cells and primary chicken cardiomyocytes. Currently, these branched peptides are tested for non-covalent delivery of aptamers and siRNA [142]. Additionally, a couple of analogues with modifications or D-amino acids were synthesised, some of which showed improved metabolic stability in cell culture, human blood [143] and also in rats [144].

#### ARF-protein derived peptide M918

M918 consists of 22 amino acids including seven arginine residues and was derived from the C-terminus of the tumor suppressor protein p14ARF by inverting a short part [54]. Its cell-penetrating properties were found only by chance when it was applied as a control peptide for activity studies of the ARF protein. FITC-labelled streptavidin was taken up by cells after formation of non-covalent complexes with 5 μM M918. The authors were able to reduce this relatively high concentration to 1 μM and achieve much higher degrees of protein uptake, when they biotinylated the peptide to improve streptavidin binding. Additionally, the peptide also delivered conjugated PNA into cells. At a concentration of 5 μM peptide/PNA-conjugate the delivery rate was significantly higher for M918 than for penetratin or TP10 [54], as well as for Tat or transportan previously analysed with the same assay [115]. Chloroquine led to a slight enhancement of the biological effect mediated by the M918/PNA-conjugate and application of the endocytosis inhibitors cytochalasin D and wortmannin suggested that micropinocytosis was involved in the cellular uptake into HeLa cells [54]. Colocalisation studies of fluorescently labelled M918 indicated that the peptide takes different entry routes in different cell lines. As no significant toxicity was detected up to concentrations of 25 μM M918, and neither any perturbation of the plasma membrane, nor an influence on the cell proliferation was measured, this peptide may have a potential for *in vivo* studies.

#### Sweet arrow peptide

The sweet arrow peptide (SAP) is derived from the proline-rich N-terminal repetitive domain of gamma-zein, a storage protein of maize which has been shown to interact with membranes [145,146]. Polyprolines adopt a well defined helical structure (polyproline II) in water, conserved even if the peptide contains only 50 % proline residues. Therefore, it is possible to generate an amphipathic helix by introducing polar amino acids at certain positions. First comparative studies showed that the sequence (VRLPPP)_3_ named SAP was most efficient for cellular uptake [70], albeit at rather high concentrations (50 μM) of the peptide. For its cellular entry a non-classical, clathrin-independent pathway through lipid raft-mediated endocytosis was proposed [141]. With the aim to increase the hydrophobicity, SAP was either modified with myristic acid [147], or one proline residue was replaced by gamma-(dimethylsila)proline [148]. As desired, these modifications led to an increase of the cellular uptake by a factor of 3 and 20, respectively. Moreover, a fully protease resistant version (i.e. all-D SAP) was synthesised in order to increase the stability of the peptide [149]. This analogue retained the cell-penetrating activity and hence was investigated in a preliminary uptake study in mice [150]. In fact, 1 h after intraperitoneal injection of 400 nmol of the peptide, uptake in leukocytes and in several organs such as kidney, liver and spleen was demonstrated. Although no bioactive cargo has been introduced so far, this study emphasised the use of D-amino acids for *in vivo* approaches which is additionally corroborated by the fact that no cytotoxicity was detected up to at least 1 mM of all SAP-analogues.

#### Dermaseptin derived peptide S4_13_-PV

S4_13_-PV is composed of 13 amino acids derived from the dermaseptin S4 peptide and the SV40 large T-antigen NLS. Dermaseptins constitute a large family of polycationic antimicrobial peptides (28–34 amino acids) which are expressed in the skin of certain tree frogs [151–153]. The peptides adopt an amphipathic α-helical structure in apolar solvents [154]. This structural feature was shown to mediate cell permeabilisation upon specific interactions with phospholipids in cellular membranes [153,155–157]. At first it had been proposed that S4_13_-PV penetrates intact HeLa cells by a non-endocytotic process and accumulates in the nuclei [71]. However, later, the peptide was reported to be internalised by endocytosis at low concentrations (0.2 to 0.4 μM) and independently of endocytosis at high concentrations (1 μM) [52]. Further biophysical studies to compare S4_13_-PV and two analogue peptides revealed that the formation of helical structures upon peptide/lipid interaction depends on the order of amino acids in the peptide [158]. The authors suggested that the higher content of helical conformation allows direct penetration of the cellular membrane. Comparative confocal microscopy of the uptake of these peptide analogues into HeLa cells confirmed the correlation of helical conformation and non-endocytotic uptake as demonstrated by the equal distribution of the peptide throughout the cytoplasm and nucleus versus a vesicular pattern observed by Mano *et al*. [159].

#### Prion protein derived peptides

Cell-penetration properties were reported for a peptide derived from the unprocessed N-terminus of mouse prion protein (mPrPp, [72]). In this study, mPrPp alone or coupled to fluorescently labelled avidin was internalised into mouse neuroblastoma cells N2a, showing both diffuse and punctuate fluorescence in the cytosol after 1 – 3 h of incubation. Recently, cellular uptake was also shown for a peptide derived from bovine prion protein (bPrPp) [73,74,160]. For both prion derived peptides, membrane perturbation effects were analysed in a number of vesicle model systems. The results indicated a peptide-induced structural defect in the membrane similar to a pore, though the overall integrity of the vesicles was not affected [73]. Furthermore, macropinocytosis was suggested as the main mechanism of uptake for bPrPp triggered by binding to cell-surface proteoglycans [161]. The prion derived peptides are known to perturb the cellular membrane, but no detailed toxicity study has been published so far. In a non-covalent complexation approach, bPrPp proved competent for the delivery of plasmid-DNA [161] as well as siRNA [76] into cells in a bioactive form.

#### Cell-penetrating pentapeptides (CPP5)

Ku70, a protein that plays an important role in DNA repair, was found to bind Bcl-2-associated X protein (Bax), a member of the Bcl-2 family of proteins, thereby inhibiting its apoptosis activating function [75]. Several pentapeptides (Bax-inhibiting peptides) derived from Ku70 of different species retained this inhibiting property and additionally featured cell-penetrating activities [162]. Human peptides with the sequences VPMLK and PMLKE as well as a sequence from the mouse (VPTLK) and the rat (VPALR) were tested for cell-penetration. To date, such pentapeptides (termed CPP5s) can be categorised as the shortest CPPs [102,163]. In order to avoid the Bax-inhibiting property, some amino acids were exchanged or inverted producing CPPs with the sequences IPMIK, KLPVM and KLPVT which were non-toxic up to concentrations of 1 mM [75]. Uptake of the CPP5s into a range of cell lines and also into primary cells was demonstrated by FACS and confocal microscopy. The mode of entry into the cell, however, as well as a potential involvement of cellular receptors is still unknown. So far, tests with endocytosis inhibitors have only been performed at an extremely high concentration of 100 μM FAM-labelled peptide showing no significant blockage of uptake. Cell culture studies at low temperatures led to a prominent reduction of cellular import but not to complete inhibition thus pointing to the involvement of both energy-dependent and -independent mechanisms. VPTLK and KLPVM were fused to the C-terminus of GFP-protein and successfully carried the cargo into HeLa cells [75].

#### Polymers and complex systems

In addition to ‘simple’ CPP-based delivery systems composed of single peptides there is a trend to develop systems of higher complexity. A main goal of such approaches is to generate nanoparticles with defined properties (e.g. size and charge distribution) as well as to provide cell-specific functionalities which are especially important for *in vivo* use. Since a comprehensive description of recent developments would be far beyond the scope of this article, we can only give a few examples. In general, there are attempts to combine peptides with cationic liposomes [94,96,164–168] or polyethyleneimine (PEI) [111]. Other applications are aimed towards the synthesis of high or low molecular weight branched polymers and/or peptides [165,169–173] or dendrimers [174–176]. Recent developments of even more complex systems are particularly promising with respect to *in vivo* delivery [177–181].

Three advanced approaches will be described in more detail below. The first is an example of a branched polymer consisting only of alternating histidines and lysines. Starting from a linear HK-peptide [182], HK-polymers with a varying number of branches were developed that showed high serum stability and efficiently delivered plasmids not only into cultured cells [170,183] but even into tumours using mouse models [184]. Interestingly, a different type of branching proved specifically advantageous for siRNA delivery [172]. A second example are Tat-grafted PEGylated nanocarriers. These carriers have been successfully applied for nucleic acid or drug delivery in several cell types and also in mouse models [178]. In order to selectively direct the delivery to cells with certain surface antigens (e. g. to tumour cells) antibodies can be conjugated to the PEG moieties (for a review on Tat-modified nanocarriers see [80]). One of the latest development of Torchilin and his group is the combination of Tat and pH-sensitive hydrazone bond-based PEG-phosphatidylethanolamine-conjugates that undergo self-assembly to form micelles in aqueous solutions. When the pH is lowered, these nanocarriers lose their PEG coating resulting in exposure of Tat. Therefore, acidic microenvironments like those found in tumours or ischemic tissues are specifically targeted and the nanocarriers and their cargo can enter the target cells [178]. Moreover, the hydrolytic stability can be adjusted by selection of particular hydrazone substituents [177]. A third example describes an equally complex and versatile delivery system, the ‘multifunctional envelope-type nano device’ (MEND). This DNA packaging approach was developed according to a rational strategy that takes into account the stability of particles, their entry into the cell as well as the release of the cargo into the cytoplasm or the nucleus. Those particles resemble very much an enveloped virus with a polycation-condensed DNA core and a lipid envelope [185]. In addition, this modular system can be changed or supplemented to meet different needs such as PEGylation for *in vivo* studies. Helper lipids like dioleoylphosphatidylethanolamine (DOPE) and cholesteryl hemisuccinate (CHEMS) can be integrated to promote endosomal release and nuclear import of the cargo, whereas the introduction of R_8_ or stearyl-R_8_ (STR-R_8_) into the envelope serves as a trigger for macropinocytosis of the particles [179,186]. Different sizes of the particles did not change the mechanism of uptake. Most importantly, the efficiency of plasmid DNA delivery of this system was comparable with that of adenovirus without inducing any cytotoxicity [179]. Additionally, successful delivery was reported for siRNA [187] and antisense oligonucleotides [188] assembled into different types of R_8_-MEND. The versatility of such a modular system was further proved in a study with the aim to specifically target skeletal muscles. For this, the heptapeptide IRQ was integrated into the MEND surface. This peptide with the sequence IRQRRRR had been discovered by *in vivo* phage display as a novel ligand for skeletal muscles and surprisingly triggered endocytosis of IRQ-MEND *via* caveolae as opposed to R_8_-MEND. The authors showed that encapsulated siRNA was imported into the cells and readily set free from endosomes only if they used a fusogenic lipid envelope for the MEND [189]. In combination with other ligands or with the tumour-homing peptides described by Enbäck *et al*. [190] recognising specific surface antigens, MEND and other delivery systems offer the possibility to develop cell-type specific therapeutic strategies.

## 5. CPP-mediate delivery of nucleic acids

In the following section we will focus on CPP-mediated cellular transport of oligomeric nucleic acids including siRNA, antisense, decoy and triplex forming oligonucleotides. Selected examples of the most recent literature will be briefly described. Additionally, examples of plasmid delivery are listed owing to their importance as vehicles for intracellular expression of small RNAs. Table 3 to 5 give an overview of all the approaches illustrated. Another important group of oligomeric nucleic acids are aptamers. These small oligonucleotides derived from an *in vitro* evolution process called SELEX are promising therapeutic and diagnostic agents. Yet, to our knowledge there are no convincing reports about peptide-mediated delivery of functional aptamers yielding observable biological effects in cellular systems.

### 5.1. Plasmids

To date there are many examples of CPP-mediated delivery of plasmid DNA into cultured cells and also *in vivo*. Due to the size of plasmids and the resulting high number of negative charges, only a non-covalent approach has proved feasible.

It has been shown that Tat peptides bind to DNA as well as other polyanions to form complexes which then interact with the membrane of different cells followed by internalisation through endocytosis [91]. According to microscopic studies, Tat peptide/DNA complexes accumulated in acidic vesicles from which they were eventually set free. Applying a similar approach, Ignatovich *et al*. [92] observed moderate reporter gene expression after incubation of cultured cells with Tat peptide/plasmid complexes. On the other hand, intravenous injection of such complexes into mice yielded only very low expression levels, predominantly in the liver. The distribution of plasmid DNA and the expression levels did not differ significantly from those obtained with naked DNA [92]. This was attributed mainly to non-specific hepatic uptake of macromolecular compounds as well as rapid clearance due to interactions with serum albumin [191,192]. Furthermore, Rudolph *et al*. [93] presented data supporting a non-covalent plasmid DNA complex formation with oligomeric Tat^47–57^. Here, dimers and trimers of Tat^47–57^ were found to be more efficient than tetramers. Another intriguing study compares the potential of high molecular weight forms of Tat and Tat peptides to form stable non-covalent cell transfecting complexes with plasmid DNA [193]. This so-called POLYTAT consists of a mixture of linear polymers of Tat peptide molecules cross linked by disulfide bridges. The diameter of such complexes was determined to be in the range of 200 nm. In contrast, complexes consisting of monomeric Tat peptides and plasmid DNA tended to aggregate into much bigger particles. POLYTAT yielded about 100-fold increased transfection rates as compared to monomeric Tat. This could be further increased by the addition of the lysosomotropic reagent chloroquine to even exceed the level of PEI-mediated transfection. The authors hypothesised that POLYTAT promotes the formation of more stable complexes that expose free basic amino acids on the surface, thereby increasing transfection efficiency [193]. This system has been further developed to form reducible layer-by-layer films with plasmid DNA in a self assembly process, which has yet to be tested for delivery [194]. Though, the idea to construct a carrier system out of several Tat peptide monomers was not new. Some years before, in 2002, it was found that branched peptides containing 8 Tat moieties had considerable transfection potential [195]. Further investigations with this branched 8Tat in the presence of chloroquine revealed that in different cell lines alternative pathways of intracellular trafficking might be relevant [196].

For R_8_-mediated plasmid delivery, low transfection efficiencies could be increased by two orders of magnitude by the attachment of a stearyl group to the N-terminus [197]. Probably due to the amphipathic character, these modified peptides reached the same level of plasmid transfection as LF and similar results were obtained for Tat^48–60^. Here, endocytosis was proposed to be the mechanism of uptake. Another example is the peptide MPG that interacts through its positively charged NLS sequence with the negatively charged plasmid DNA. Such peptide/nucleic acid complexes have been reported to be taken up efficiently by mammalian cells [130]. The non-covalent complexation of the prion protein derived peptide bPrPp with plasmid DNA led to endocytotic uptake of the particles and protein expression from the plasmid. The expression levels, however, were much lower than measured after LF-mediated transfection even with peptide concentrations up to 29 μM [161]. Similarly, none of the branched human calcitonin derivatives hCT^9–32^-2br and hCT^18–32^-k7 could reach the transfection rate of Lipofectamine™ 2000 (LF2000) in the cell lines HEK 293, HeLa, MCT-7, COS-7 and SK-N-MC or in primary cells. Although the branched peptides were more efficient than Tat^48–60^, the transfection rate was only about 40 % of that seen with LF2000, even when tested in the presence of 125 μM chloroquine and at the optimal peptide/plasmid charge ratio (30:1). Fluorescence microscopic localisation studies led to the conclusion that both branched CPPs delivered the plasmids into all cells *via* endocytosis with an unsatisfying extent of endosomal escape. Interestingly, the protein expression level in neuroblastoma cells was 1.5-fold higher than after LF2000-mediated transfection [142].

A novel arginine-grafted polymer for gene delivery with low cytotoxicity was developed by Kim *et al*. [198]. This copolymer consists of two PAMAM moieties separated by PEG and flanked by arginine residues. It forms nanosized polyplexes with plasmids that were delivered into various cell lines. The precursor polymer lacking the arginines showed only a minor capability to promote gene expression from the delivered plasmid, presumably because it cannot escape from endosomes. In contrast to this, transfection with the arginine modified polymer led to significantly enhanced gene expression. Based upon extensive studies with inhibitors of endocytosis, the authors suggested that this delivery system enters the cell *via* a combination of pathways [198].

From the literature currently available, it seems that for *in vivo* studies CPP-based polymers or complex systems are preferable as opposed to simple CPP approaches. To give an example, the histidine/lysine-polymer H2K4b proved to be useful for plasmid delivery into several cell lines as well as in mouse models. In comparison to other versions, this 4 branched system had the highest efficiency [183]. *In vivo*, after systemic administration of a luciferase plasmid in mice with a xenograft tumour, gene expression was mostly detected in lung and spleen, in addition to tumour tissue. In two models with different growth rates the tumour size was significantly reduced when the transfected plasmid encoded antitumour genes [184].

As it has been already mentioned in the chapter ‘polymers and complex systems’, the ‘multifunctional envelope-type nano devices’ (MEND) mimic virus particles [185]. In order to compare the transfection efficiency of different MEND types for gene delivery, DNA coding for luciferase was used as a cargo. Out of several different combinations of peptides and lipids tested in cell culture the highest luciferase activity was achieved with MEND3, which are poly-L-lysine/DNA particles coated with endosomolytic lipids and stearyl-R_8_ (DOPE/CHEMS/STR-R_8_). Most impressively, the efficiency of this transfection method was comparable with that of adenovirus without inducing any cytotoxicity. Additionally, the authors present a successful gene delivery into hair follicles of mice after topical application at a rate of transfection much higher than achieved with LF [179].

### 5.2. Antisense oligonucleotides

By far the most common use of oligonucleotides as inhibitors of gene expression is the so-called antisense approach (for a review see: [199,200]). Antisense oligonucleotides are complementary to the RNA of interest, therefore specificity is mediated through Watson-Crick base pairing of the oligonucleotide with the target RNA. The three principle ways that antisense oligonucleotides have been used to disrupt protein production are: (I) the oligonucleotide/RNA duplex may form a substrate for endogenous RNase H, leading to mRNA cleavage; (II) the oligonucleotide/RNA duplex may prevent the productive assembly of the ribosomal complex or arrest a ribosomal complex already engaged in translation, in both cases affecting protein biosynthesis; (III) the oligonucleotide/RNA duplex may alter pre-mRNA splicing in the nucleus. Early studies demonstrating antisense oligonucleotide-mediated effects (i.e. inhibition of neurite growth *via* downregulation of the amyloid precursor protein [14] or cell death after downregulation of a Cu/Zn superoxide dismutase [201]) were performed with penetratin-DNA conjugates. In addition, conjugates of Tat peptide or penetratin with phosphorothioate modified oligonucleotides were effective in antisense inhibition of P-glycoprotein expression, a membrane ATPase associated with multidrug resistance in tumour cells [86]. However, most peptide-based delivery studies of antisense oligonucleotides were conducted with peptide nucleic acids (PNAs) [17,85,105,112,202–205]. PNAs are nucleic acid mimics in which the ribose-phosphate skeleton has been exchanged with a simpler polyamide backbone [206]. PNAs bind to both single-stranded DNAs and RNAs with high affinity and sequence specificity. Furthermore, PNAs bind to double-stranded DNAs through the unique mechanism of so-called strand invasion. In addition to their remarkable hybridisation properties, PNAs are resistant to nucleases and proteases because they lack anomeric carbon atoms and standard amino acids. Owing to their inability to activate RNase H, in contrast to unmodified antisense oligonucleotides, PNAs merely act as a steric block.

The first study showing CPP-mediated antisense activity for a PNA was conducted by Pooga *et al*. [112]. Here, a suppression of the galanin receptor expression in cell culture as well as in a rat model was achieved by coupling a corresponding 21mer PNA to penetratin or transportan. Additionally, it was reported that a 16mer PNA coupled to transportan and targeted to the HIV-1 transactivator responsive region (TAR) RNA was efficiently internalised into cultured cells [203]. Examination of the functional efficacy of the PNA-transportan conjugate in cell culture using a luciferase reporter gene assay revealed a significant inhibition of Tat-mediated transactivation of HIV-1 long terminal repeat. Furthermore, the conjugate substantially inhibited HIV-1 production in chronically HIV-1 infected H9 cells [203].

More recently, a PNA with an almost identical sequence, disulfide-linked to either transportan or the chimeric peptide R_6_-penetratin was shown to exhibit dose-dependent inhibition of Tat-mediated transactivation in a HeLa cell assay when incubated for 24 h [207]. When chloroquine was co-administered, transactivation activity was already reached within 6 h. Interestingly, fluorescein-labelled stably linked conjugates of Tat, transportan or TP10 with the same PNA were inactive when delivered alone, but attained transactivation inhibition in the presence of chloroquine. The data presented indicate that a cleavable bond is not essential for activity in this assay. Moreover, confocal microscopy showed that fluorescently labelled CPP-PNA conjugates were sequestered in endosomal or membrane-bound compartments of HeLa cells, which varied in appearance depending on the CPP. Coadministration of chloroquine was seen in some cases to release fluorescence from such compartments into the nucleus, but with different patterns depending on the CPP. These findings of Turner *et al*. [207] are inconsistent with observations of Tripathi *et al*. [205]. The latter suggested a non-endocytotic pathway for the uptake of disulfide-linked conjugates of anti-TAR PNA with several CPPs as illustrated by flow cytometry analysis. Additionally, an inhibitory effect on HIV-1 replication with IC_50_ values in the submicromolar range as well as viricidal activity in the low nanomolar range for the conjugates tested was reported. Very recently, they evaluated pharmacokinetic properties of the anti-TAR PNA-penetratin conjugate in Balb/C mice and concluded that the construct should be nontoxic in the concentration range predicted for a future therapeutic use [107].

Using an improved purification protocol, Turner *et al*. [208] synthesised several CPP-conjugated 2′-*O*-methyl RNA oligonucleotides (OMe) and OMe/locked nucleic acid (LNA) mixmers as well as OMe-phosphorothioate RNA oligomers targeted to HIV-1 TAR. Although all oligonucleotides had previously shown activity in the HIV-1 transactivation assay after cationic lipofection [209], no activity was detectable for the highly pure conjugates. In agreement with this, only vesicular uptake, but no nuclear import was observed by confocal microscopy. In order to generate a net positive charge of the conjugates, additional basic amino acids were introduced into the peptide sequence, which did not enhance bio-availability of the oligonucleotides either. Interestingly, the rate of uptake was dramatically enhanced by addition of free CPP to the conjugates, though still no biological activity was observed, indicating a possible lack of endosomal escape [208]. The authors suggested that these free CPPs form complexes with CPP-cargo conjugates, which play a significant role in the uptake process and concluded that care has to be taken during conjugate purification. Furthermore, this study shows that the uptake pattern strongly depends on the cell line analysed.

Astriab-Fisher *et al*. [87] described delivery of OMe RNA phosphorothioate oligonucleotides linked *via* disulfide bridge to Tat peptide and penetratin. As a biological readout a splice correction assay [210] was applied. This assay uses antisense-mediated rescue of an introduced aberrant splice site which otherwise leads to an inactive reporter enzyme, in this case luciferase. Such an approach is particularly interesting since the reporter gene activity is turned up rather than turned down upon application of the appropriate antisense oligonucleotide. Thus, negative effects caused by experimental constraints leading to reduced protein expression, which are not due to the applied nucleic acid, will not cause false interpretation of the experimental data. The CPP-oligonucleotide conjugates progressively entered cells in a matter of hours and were detected both in cytoplasmic vesicles and in the nucleus [87]. The conjugates targeted to the aberrant splice site, but not the mismatched controls, caused an increase in luciferase activity in a dose-responsive manner. These findings are in contrast to the results of Turner *et al*. [208] described above, who could not find a biological effect. This discrepancy may simply be due to differences between the two biological assays.

Phosphorodiamidate morpholino oligomers (PMOs) are similar to DNA with two major structural differences: the negatively charged phosphorodiester internucleoside linkage in DNA is replaced by the neutral phosphorodiamidate linkage and the five-membered ring of deoxyribose in DNA is replaced by the six-membered ring of morpholine. The uncharged and hydrophilic PMOs are highly resistant to enzymatic degradation. Using the same splice correction assay as described above, Moulton *et al*. [211] could show that missplicing of pre-mRNA was corrected upon addition of a R_9_F_2_-PMO conjugate into cell culture medium at low micromolar concentrations. Delivery of PMOs to the cell nucleus and cytosol required conjugation rather than complexation of peptides to PMOs. Furthermore, the arginine-rich peptide R_9_F_2_ showed higher transfection rates than conjugates with Tat peptide, penetratin or a Tat peptide analogue. The comparison of conjugates with various linkers revealed increased antisense activity of R_9_F_2_-PMO conjugates with longer spacers whereas variation in conjugation chemistry did not result in any differences [211]. Additional studies of the same group show inhibition of coronavirus, flavivirus, Dengue virus and West Nile virus replication by CPP-mediated antisense PMO delivery [212–216].

Besides the addition of chloroquine, different endosome disrupting strategies have been evaluated using the splice correction assay, for example cotreatment with endosome-disruptive peptides [115] or photochemical internalisation [129]. However, the most promising results, especially concerning future *in vivo* applications of steric block oligonucleotides, have been achieved with two newly developed derivatives of classical CPPs (reviewed in [217]). The modification of oligoarginines with non-natural, uncharged amino acids [218] led, amongst others, to the peptide (R-Ahx-R)_4_, in which Ahx represents a six-atom aminohexanoic acid spacer. Abes *et al*. demonstrated that in contrast to Tat or oligoargine, PMO-conjugates of this peptide led to dose-dependent splice correction at low micromolar concentrations in the absence of endosomolytic agents. The underlying mechanism for this superior activity is not clear yet, as the uptake of (R-Ahx-R)_4_ constructs was less efficient than the uptake of Tat or oligoarginine constructs and also involved endocytotic routes [219]. The second peptide is a derivative of penetratin, to which six arginine residues were added at the N-terminus (R_6_Pen). R_6_Pen-PNA conjugates were shown to inhibit HIV-1 Tat-dependent transactivation [207] as well as promote efficient splice correction, in both cases at low concentrations and in the absence of endosomolytic agents [109]. Again, uptake of R_6_Pen-conjugates seemed to involve endocytosis and there was nearly a difference in splice correcting activity regardless of the nature of the linker used for conjugation, like a stable thioether versus a reducible disulfide linker [217]. CPP-PMO or -PNA-conjugates are beginning to advance from research tools to therapeutic application. *In vivo* efficacy has already been demonstrated in mouse models for muscular dystrophy and coronavirus infection using another variant of the (R-Ahx-R)_4_-peptide described above ([220] and references therein).

In this regard, another very interesting approach is followed by Morris *et al*. [134] using negatively charged PNA-like DNA mimics called HypNA-pPNA, which consist of phosphonate analogues of PNA and PNA-like monomers on the basis of trans-4-hydroxyl-L-proline [221]. Based on a previous peptide from the Pep family [133], the group designed a new CPP, Pep-3, which forms stable non–covalent complexes with charged as well as uncharged PNAs [134]. Efficient cellular uptake of an antisense HypNA-pPNA was demonstrated *via* downregulation of cyclin B1 in HeLa cells as well as in suspension and primary cells. Furthermore, a mouse xenograft tumour model of human prostate carcinoma was used to analyse *in vivo* delivery of Pep-3/HypNA-pPNA complexes. Intravenous administration reduced tumour growth by approximately 20 %, but after intratumoural injection, a specific and concentration-dependent inhibition of tumour growth up to 90 % was detected, which could be further improved by stabilising the complex through PEGylation at the N-terminus.

MicroRNAs (miRNAs), a conserved class of small non-coding RNAs, participate in the post-transcriptional regulation of many cellular processes and are also involved in the emergence of tumours or metabolic diseases [222]. Specific miRNA-silencing can be achieved through the administration of antisense oligonucleotides, so called antagomirs [223]. Along these lines, Fabani *et al*. investigated the blocking activity of an anti-miR-122 PNA conjugated to the R_6_-modified penetratin described above [108]. Knockdown of miR-122 to a very low level after incubation with the inhibitory construct was verified by Northern blot analyses as well as by up-regulation of mRNAs normally negatively regulated by miR-122. Strikingly, incubation with an unconjugated PNA which was only modified with 4 lysine residues, led to a complete knockdown of miR-122. Cellular uptake of similar PNA-Lys oligonucleotides had been observed earlier [207,219], but in these cases was apparently not sufficient to obtain the desired biological activity in the nucleus.

### 5.3. Transcription factor decoy oligonucleotides and triplex forming oligonucleotides

Double-stranded transcription factor decoy oligonucleotides are a powerful tool to modulate gene expression. Decoys compete with response elements within the promoter regions of genes that bind transcription factors.

Coupling a NFκB specific decoy to either transportan or TP10, Fisher *et al*. [113] could show efficient cellular translocation of this construct going along with an inhibition of interleukin(IL)-1β-induced NFκB activation and NFκB downstream effects. The coupling was achieved by hybridising a PNA to the decoy, which contained a corresponding single strand overhang, while the PNA itself was linked to the CPP *via* a disulfide bridge. Recently, the authors applied this principle to a model of Alzheimer'sdisease in primary rat glial cells which are much harder to transfect. This resulted in 80 % inhibition of the NFκB binding activity leading to a decrease of the IL-6 mRNA expression by 50 % [224].

Using a similar approach, El-Andaloussi *et al*. [225] presented uptake experiments comparing the effect of a Myc decoy either covalently or non-covalently attached to TP10. The Myc protein had been shown to be overexpressed in 50 – 60 % of human tumours known today and, being well characterised, represents a favourable target for anticancer therapies. Besides the biological effect, the intracellular amount of decoy was determined by fluorescence measurement. Compared to non-covalent complexation of TP10 and decoy, the oligonucleotide was taken up about 50-fold less when hybridised to the TP10-PNA conjugate. However, this did not correlate with biological activity, as the conjugate was just 2-fold less effective. Intriguingly, various endocytosis inhibitors had no effect on the rate of internalisation suggesting that peptide/cargo complexes bypass the endosomal pathway to a large extent. Therefore, the low bio-availability must be caused by other unknown intracellular processes.

Triple helix-forming oligonucleotides (TFOs) bind as a third strand in the major groove of duplex DNA to form triplex DNA in a sequence-specific manner (for a review see: [226]). Attractive applications of this TFO approach include not only inhibition of the target gene, but also induction of point mutations at predetermined sites. A TFO coupled to penetratin by disulfide linkage was shown to internalise into cells [227]. In this approach the supFG1 reporter gene, encoding an amber suppressor tRNA, was used as target and the number of mutations induced by the TFO was determined with the help of an *E. coli* strain expressing β-galactosidase with an amber mutation. After treatment of cultured mouse cells, the rate of mutation was documented to be enhanced by a factor of 20 compared to basal levels. Confocal microscopy studies confirmed uptake into the nucleus and also revealed that the sequence of the oligonucleotide cargo affected the efficiency of delivery and the pattern of intracellular distribution (e. g. a higher GC-content seemed to reduce nuclear accumulation).

### 5.4. siRNA

In addition to the antisense applications described above, siRNAs represent a further valuable antisense tool to inhibit the expression of a target gene in a sequence-specific manner. These small RNA molecules induce a process termed RNA interference (RNAi) resulting in mRNA degradation (for a review see: [228,229]).

The first study about CPP-mediated delivery of siRNA was published in 2003 by Simeoni *et al*. [131]. Here, siRNAs were non-covalently complexed with the peptide MPG leading to a strong down regulation of the target protein. Interestingly, a mutation in the NLS sequence of the carrier peptide (MPGΔ^NLS^) resulted in a slight increase of the RNAi effect. When siRNA was associated with MPG at a 1:10 ratio of negative to positive charges and applied to Cos-7 or HeLa cells, a decrease of about 80 % in luciferase activity was detected. This effect was further enhanced to about 90 % by MPGΔ^NLS^ [131].

Other studies describe covalent attachment of cargo and carrier. In one approach, anti-GFP or anti-CDK9 siRNA were cross-linked to Tat^47–57^, but significant down-regulation of the target protein could only be observed for high concentrations of nucleic acids (about 300 nM, [90]). Simple mixing of siRNA and Tat peptide did not lead to any measurable RNAi effect. LF- or Tat^47–57^-mediated transfection resulted in a perinuclear localisation of siRNA. In contrast, fluorescently labelled Tat^47–57^ without cargo was mainly found in the nucleolus. A significant increase in the rate of uptake of siRNAs targeted against luciferase or GFP could be observed after disulfide coupling the 5′-end of the sense strand to penetratin or transportan [104]. Compared to LF2000, slightly higher levels of transfection were achieved. Interestingly, after LF2000-mediated transfection, basal luciferase activity returned to normal levels one day earlier than after CPP-mediated transfection although the same concentration of siRNA was applied.

A remarkably strong RNAi effect in hard to transfect primary neuronal cells was reported by Davidson *et al*. [230]. Here, siRNAs directed against several endogenous proteins were coupled to penetratin *via* a disulfide bond. The observed down regulation of the target proteins after peptide-mediated siRNA delivery was found to be far more effective compared to LF2000. This in part was attributed to the toxicity of the lipids.

Dowdy and his group [231] presented a rather critical point of view referring to previous studies with CPP-siRNA-conjugates. They claim that the successful delivery described therein is solely the result of excess free peptide which leads to additional complexation and thereby cellular import of the siRNA. This is in accordance with Turner *et al*. [208], who were the first to observe that careful purification of CPP-antisense-conjugates abrogates their biological effect.

Lundberg *et al*. [76] rationally modified penetratin to form a CPP (termed EB1) with improved endosomolytic properties. They achieved a pH-dependent conformational change of the peptide to a higher degree of helicity by the replacement of two basic amino acids with histidines and the N-terminal addition of six amino acids. In this study, several CPPs were compared in a non-covalent approach by measuring the overall cellular uptake *via* fluorescence and biological effect of siRNA targeted to the luciferase mRNA. Penetratin- as well as TP10-mediated transfection did not lead to any silencing of luciferase gene expression, despite high amounts of intracellular siRNA [76] and in contrast to previous achievements with siRNA-penetratin-conjugates [230] or TP10/DNA-complexes [225]. EB1 showed improved delivery with a reduction of luciferase activity to approximately 50 % at 100 nM siRNA. The peptide also induced RNAi in HepG2 cells but to achieve this, the transfection protocol had to be changed, i.e. the preincubation volume had to be increased, emphasising differences in uptake properties of different cell lines. As it had been described earlier, that addition of a pH-sensitive peptide derived from haemagglutinin (HA2) can promote endosomal escape [43], the authors linked HA2 to penetratin [76]. It turned out that although HA2-penetratin improved the silencing effect when coincubated with penetratin, EB1 was more potent than this combination of peptides. Together with confocal microscopy studies the authors concluded that the lack of biological effect after penetratin-mediated siRNA delivery is due to a lack of endosomal escape and that EB1 has a superior endosomolytic activity in comparison to HA2-penetratin. In addition to EB1, MPGΔ^NLS^ and bPrPp were analysed in this study. For all three peptides, a much lower silencing effect was seen compared with LF2000 after transfection of 100 nM siRNA [76].

In a recent study Nakamura *et al*. reported condensing of siRNA with 3 types of positively charged agents: poly-L-lysine, stearyl-R_8_ (STR-R_8_) and protamine [187]. These siRNA cores were packaged into a R_8_-grafted lipidic envelope to form R_8_-MEND. It was shown that R_8_-MEND particles with STR-R_8_-condensed siRNA yielded the smallest complexes and the most efficient silencing effect in HeLa cells. At a concentration of 60 nM siRNA 80 % of gene silencing was achieved. Transfection with siRNA/STR-R_8_ cores lacking the lipidic envelope did not lead to any biological effect, probably due to inefficient endosomal escape [187]. In spite of this, another study provides evidence that siRNA/STR-R_8_ particles alone can elicit RNAi in primary rat neuron cells [232].

A promising result with a prospect for cell specific siRNA delivery was presented by Leng *et al*. [172]. In an attempt to optimise a branched histidine/lysine-polymer for siRNA delivery, the authors found that different 8 branched versions (H3K8b) yielded up to 80 % knockdown of the target gene in several cell types. A 4 branched H2K4b, on the other hand, turned out to be a suitable carrier for plasmids [184] but not for siRNA. Structure-function studies revealed an important role of the composition of the histidine-rich domain as well as its position within the peptide and the branches for siRNA delivery, whereas size and surface charge did not have any effect. Furthermore, the toxicity was much lower than for the commercial cationic lipids Oligofectamine and LF2000 [172]. The attachment of the tripeptide RGD, an integrin-ligand, only slightly enhanced siRNA delivery, but turned this carrier into a cell-specific system [172].

As one of the first groups to report on Tat^48–60^- or penetratin-mediated siRNA delivery *in vivo*, Moschos *et al*. showed, that intratracheal administration of the conjugates did not lead to any intensification of the knockdown of the target gene p38 mitogen-activated protein kinase in mouse lungs in comparison to unmodified non-formulated siRNA [106]. They give an overview of *in vivo* studies with siRNA alone or with several non-peptidic carrier systems showing, that even unmodified and non-formulated siRNA can exert a significant silencing effect [233]. Strikingly, it was found that the peptides alone triggered a detectable decrease in target gene expression and that the penetratin-conjugate induced elevated levels of the immune markers IFN-α, TNF-α, and IL-12p40 in lung tissue [106]. This emphasises that a conclusion cannot easily be extrapolated from *in vitro* experiments and applied to the *in vivo* situation and that the experimental conditions have to be carefully controlled.

Meanwhile, CPP-mediated siRNA delivery has been shown to be successful at least in some *in vivo* studies. In one of them, subcutaneous injections of siRNA non-covalently complexed with cholesteryl oligo-D-arginine (Chol-R_9_) in a mouse model successfully targeted the angiogenic growth factor VEGF (vascular endothelial growth factor). Seventeen days post-administration, target protein expression in the tumour decreased to approximately 40 %. More importantly, a tumour regression by a factor of 7 was measured [121].

It is long known, that a peptide derived from rabies virus glycoprotein (RVG) interacts specifically with the nicotinic acetylcholine receptor (AchR) on neuronal cells to enable viral entry. Only recently, Kumar *et al*. used this specificity for a delivery approach into the brain [234]. Remarkably, the biotinylated form of the 29-amino-acid peptide (YTIWMPENPRPGTPCDIFTNSRGKRASNG) was taken up by neuronal cells in the brain after injection into mice. In order to transport nucleic acids with this vehicle, R_9_ was conjugated to RVG peptide. Systemic treatment of mice with siRNA in a non-covalent complex with this modified peptide promoted a highly specific cellular import of siRNA only into cells expressing AchR. Even more important, an antiviral siRNA treatment resulted in successful protection of mice against encephalitis caused by Japanese encephalitis virus (JEV) [234]. This is the first study to report on a non-toxic method to deliver siRNA across the blood brain barrier which could help to circumvent dangerous and ineffective injections into the brain. To date it presents one of the most promising delivery approaches which might be expandable to other *in vivo* applications.

So far, no CPP-mediated transfection of miRNAs has been reported on. It was only shown that a primary microRNA (pri-miRNA) comprising 183 nucleotides has been transferred into the nucleus of HeLa cells after complexation with the so called reducible copolypeptide (rCPP) [181]. This linear delivery system is composed of the lysine-containing histidine-rich peptide (HRP) and the SV40 large T-antigen NLS randomly connected *via* disulfide bonds and was developed by Manickam *et al*. for plasmid delivery [180]. The intranuclear processing of pri-miRNA into mature miRNA was possible only if the polymers contained a sufficient portion of NLS moieties. The authors also studied the efficiency of rCPPs for cellular delivery of siRNA. It turned out that a low NLS-content in the polyplex was favourable for posttranscriptional cytoplasmic RNAi, whereas a higher NLS-content promoted nuclear delivery. Thus, siRNA-mediated promoter silencing was enabled which even excelled the efficiency of the commercial transfection reagent TransIT-TKO [181].

From these examples of CPP-mediated delivery of nucleic acids it becomes obvious, that in most cases a delivery system must be adjusted to the sort of nucleic acid to be transfected. The enormous difference in size and number of charges between oligonucleotides and plasmid DNA leads to different mechanisms of non-covalent particle formation. This partly can explain cargo-dependent discrepancies that have been found also for lipidic carrier systems [235].

## 6. Cellular uptake versus functional effects of the nucleic acid cargo

To efficiently exert their activity, nucleic acid-based tools and drugs have to reach their cellular targets after gaining entry to the cell (for a recent review see [237]). To claim ‘proof of principle’ for a particular CPP approach, it might be sufficient to just measure uptake of a given functional nucleic acid cargo (e.g. siRNA, aptamers or antisense RNA). However, it does not provide any information about the biological activity of the cargo. Thus, a side-by-side analysis of cargo internalised and the corresponding biological effect is required to determine the overall efficacy of such a system. We have adapted a highly sensitive method described by Overhoff *et al*. [238] for the quantification of siRNA, enabling us to detect intracellular siRNA amounts down to ≥ 10 copies per cell. The method is based on the liquid hybridisation of a radioactively labelled probe with the corresponding antisense strand of the siRNA in cellular lysates. Additionally, a stringent washing procedure is absolutely required. As already outlined above, complexes strongly attached to the cell surface are often an underestimated source of misinterpretation of the true amount of cargo taken up by cells. By implementing a heparin wash, we could reduce the overall amount of allegedly ‘intracellular’ siRNA by about 90 % [236]. This result impressively shows the importance of such a procedure.

As opposed to the majority of CPP applications reported, which rely on covalent linkage of carrier and cargo, we used in our studies a peptide termed MPGα, a derivative of the original MPG peptide described by Morris and coworkers [19]. This peptide forms highly stable non-covalent complexes with nucleic acids. MPGα differs from MPG by six amino acids in the hydrophobic part. These changes result in an alteration of the overall structure of the peptide towards a higher tendency of adopting a helical conformation [68]. Accordingly, the two peptides behave differently with respect to their interaction with artificial lipids as well as *Xenopus* oocytes [239] and should not be confused as, most probably, their exact mechanism of uptake is not the same.

**Figure 5.**
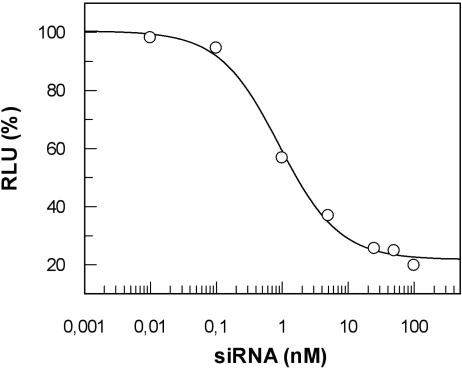
**IC_50_ of luciferase targeted siRNA delivered by complexation with MPGα into ECV304 cells.** 24 h before transfection ECV304-cells were seeded into a 96 well plate (1×10^4^ cells per well). siRNA and MPGα were mixed in Opti-MEM^®^ I (4.2 μM final concentration of peptide) and incubated for 5 min at room temperature. Cells were overlaid with the complexes for 4 h followed by addition of medium supplemented with 10 % FCS. 24 h after transfection, luciferase activity was measured in a plate reader and cell numbers were normalised with the help of fluorescein diacetate. The IC_50_-value of 0.8 nM was calculated using the program Grafit.

The delivery of anti luciferase siRNA/MPGα complexes into HeLa or ECV304 cells yielded an IC_50_ of about 0.8 nM (Figure 5). Although the number alone is already quite impressive and convincingly shows that this approach works very well in cell culture experiments, it does not provide information about the percentage of bio-available siRNA taken up. Comparing the amount of extracellular RNA with the amount of RNA internalised, it becomes obvious, that less than 1 % entered the cells. However, even more remarkable are the data derived from a comparison with microinjection experiments [236]. These indicated that less than 1 % of the molecules taken up enter the RNAi machinery. Thus the vast majority of molecules probably are in a physical state, which is not suitable for triggering RNAi.

**Figure 6.**
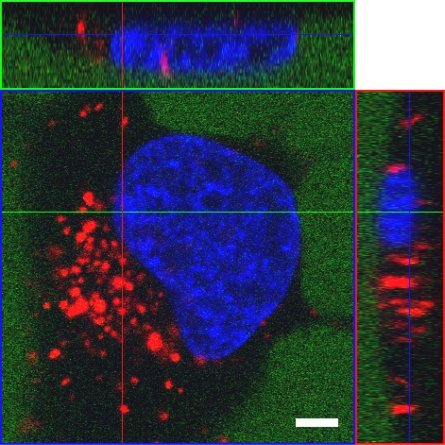
**CLSM analysis of unfixed HeLa cells after transfection with MPGα/RNA aptamer complexes.** Cells were incubated in Opti-MEM^®^ I for 3 h with MPGα/RNA complexes (5 μM peptide and 180 nM RNA). The RNA was 5'-labelled with Cy3. After a wash step with PBS, cells were treated with a solution of heparin (15 units/mL) in Opti-MEM^®^ I for 30 min, washed with Opti-MEM^®^ I, stained with Hoechst 33342 and overlaid with Opti-MEM^®^ I containing 50 mM HEPES, pH 7.4. The extracellular space was stained by adding carboxyfluorescein to the medium. Microscopical analysis was performed with a confocal laser scanning microscope (LSM 510, Carl Zeiss). White bar: 5 μm.

Confocal microscopy studies with fluorescently labelled oligonucleotides reveal a punctual cytoplasmatic distribution of the molecules after MPGα mediated uptake (Figure 6). Together with the data described above and data concerning the effect of specific inhibitors/effectors of different endocytotic pathways [236], this strongly indicates endocytosis to be involved in uptake. Accordingly, if it would be feasible to liberate the siRNA molecules trapped in vesicular compartments, one would be able to dramatically boost the overall efficacy of this approach. Though others have reported similar findings, our study for the first time puts in numbers how much room for improvement there actually is. Altogether, the data presented clearly show the enormous potential of this particular CPP-mediated cellular delivery of siRNA, which likely is true to various degrees for other similar approaches as well, but at the same time reveal the problems to be resolved in the future.

## 7. Conclusion

Owing to recent critical evaluations of the mechanisms underlying the cellular translocation of CPPs it had become apparent that technical shortcomings caused false assumptions, which led to an overestimation of translocation efficiencies as well as misinterpretations concerning the cellular uptake mechanism. As a consequence, efforts to improve CPP transfection efficiency might have been hampered or even worse, went in the wrong direction. Meanwhile many problems have been identified and the majority of present studies implement latest technical achievements by combining a variety of complementary methods. Today, there is considerable evidence that endocytosis is involved in the internalisation process of various CPPs. As more information about the exact mechanism of uptake is collected with an increasing rate, significant improvements of such delivery systems can be expected in the near future.

On the other hand, currently available data are based on studies using a variety of different cell lines and techniques, which renders a direct comparison of different CPPs impossible. It therefore would be highly desirable to establish standardised reporter systems, which would facilitate a quantitative analysis of exceedingly diverse approaches. Furthermore, it has been shown that even minor changes of the physical state of a CPP (e.g. exchange of certain amino acids) can alter translocation properties significantly. This particularly holds true for the attachment of large cargo molecules. Thus, it might not be possible to generalise results obtained with a given CPP, and it might be necessary to characterise each carrier/cargo complex individually. If CPPs are intended to be used for therapeutic purposes in the future, it is essential to focus on the attachment of functional cargos and analyse their biological effects inside the cell. Data from our lab clearly show that uptake and biological activity of a functional cargo is everything but the same. Therefore, a quantitative comparison of cargo taken up and functionally active cargo is an essential requirement in order to improve therapeutic efficacy. Just looking for efficient internalisation is not sufficient. As a prerequisite, there is a need for sensitive and easy to handle reporter systems in combination with a sensitive method to quantify intracellular amounts of cargo like the one for siRNA briefly described above. Data established with this system for MPGα-mediated delivery of siRNA as well as a number of other recent studies reviewed here strongly suggests endosomal accumulation of the cargo and not uptake *per se* to be the bottleneck of this approach. Thus, resolving the problem of endosomal escape currently is the main challenge for this CPP-system.

In conclusion, current reports provide increasing evidence that peptides represent a promising alternative to viral and lipid-based nucleic acid delivery systems. After two decades of intensive research, we now can chose from a constantly growing arsenal of different peptide-based transfection systems each suitable for a particular application. For nucleic acid delivery *in vitro* simple peptide carriers are probably the first choice. Here the described non-covalent approach offers the degree of flexibility required for basic research applications where experimental conditions may change frequently. For *in vivo* applications a trend towards more complex and elaborate systems (see chapter ‘polymers and complex systems’) can clearly be seen. Such approaches are most promising to individually resolve important issues like undesired degradation or clearance from the body and above all target cell specificity.

## Figures and Tables

**Table 1.** Sequences of selected classical CPPs.

Peptide	Sequence	Reference
Tat^48–60^	GRKKRRQRRRPPQ	[20]
penetratin (Antp^43–58^)	RQIKIWFQNRRMKWKK	[63]
transportan	GWTLNSAGYLLGKINLKALAALAKKIL	[64]
TP10	AGYLLGKINLKALAALAKKIL	[65]
Oligoarginine (R_8_)	RRRRRRRR	[22,66]
MAP	KLALKLALKALKAALKLA	[67]
MPG	GALFLGFLGAAGSTMGAWSQPKKKRKV	[19]
MPGα	GALFLAFLAAALSLMGLWSQPKKKRKV	[68]

**Table 2.** Sequences of selected new CPPs.

Peptide	Sequence	Reference
hCT^9–32^-br	LGTYTQDFNK*FHTFPQTAIGVGAP (-AFGVGPDEVKRKKKP; attached to K*)	[69]
SAP	(VRLPPP)_3_	[70]
S4_13_-PV	ALWKTLLKKVLKAPKKKRKV	[71]
mPrPp	MANLGYWLLALFVTMWTDVGLCKKRPKP	[72,73]
bPrPp	MVKSKIGSWILVLFVAMWSDVGLCKKRPKP	[73,74]
M918	MVTVLFRRLRIRRACGPPRVRV	[54]
CPP5s	VPMLK, PMLKE (human), VPTLK (mouse), VPALR (rat)	[75]
EB1	LIRLWSHLIHIWFQNRRLKWKKK	[76]

**Table 3.** Examples for delivery of plasmids.

Cargo	CPP/delivery system	proposed uptake mechanism	Reference
plasmid DNA	MPG	non-endocytotic	[130]
plasmid DNA	R_8,_ stearyl-R_8_, Tat^48–60^	endocytotic	[197]
plasmid DNA	branched 8Tat peptide	endocytotic	[195]
plasmid DNA	Tat^48–60^-peptide	endocytotic	[91]
plasmid DNA	Tat^47–57^-oligomers	endocytotic	[93]
plasmid DNA	Tat^47–57^	endocytotic	[92]
plasmid DNA	branched 8Tat peptide	endocytotic (dependent on cell line)	[196]
plasmid DNA	POLYTAT	endocytotic	[193]
Plasmid	bPrPp	endocytotic	[161]
DNA/looped DNA			
plasmid DNA	hCT^9–32^-2br, hCT^18–32^-k7	endocytotic	[142]
plasmid DNA	R-PAMAM-PEG-PAMAM-R dendrimer	endocytotic	[198]
plasmid DNA	HK-polymer	endocytotic	[183,184]
plasmid DNA	R_8_-MEND3	endocytotic	[179]

**Table 4.** Examples for delivery of oligonucleotides and derivatives thereof.

Cargo	CPP/delivery system	proposed uptake mechanism	Reference
DNA oligonucleotide	MPG	non-endocytotic	[19]
antisense PNA	penetratin, transportan	n. d.	[112]
antisense PNA	transportan	n. d.	[203]
antisense PNA	Tat^48–60^, penetratin, transportan analogues	non-endocytotic	[205]
antisense PNA	Tat^48–58^, penetratin, transportan analogues, R_9_F_2_, R_6_-penetratin and further peptides	endocytotic (transportan: possibly non-endocytotic)	[207]
antisense PMO	R_9_F_2_ (non-covalent + covalent), Tat peptide, penetratin (covalent)	n. d.	[211]
antisense PMO	(R-Ahx-R)_4_	endocytotic	[219]
antisense PNA	R_6_-penetratin	endocytotic	[109]
antisense PMO	(R-Ahx-R)_4_AhxB	n. d.	[220]
HypNA-pPNA	Pep-3	n. d. (proposed non-endocytotic)	[134]
Antagomir	R_6_-penetratin	n. d.	[108]
antisense 2'-OMe phosphorothioate RNA oligonucleotides	Tat^49–60^, penetratin	endocytotic	[87]
antisense RNA oligonucleotide analogues	Tat^48–58^, penetratin, R_6_-penetratin, transportan, R_9_, R_9_F_2_ and further peptides	endocytotic	[208]
decoy	PNA-coupled transportan or TP10	n. d.	[113,224]
decoy	PNA-coupled transportan or TP10 + NLS	non-endocytotic, to small extent endocytotic	[225]
TFO	penetratin	non-endocytotic endocytotic pathway not excluded	[227]

**Table 5.** Examples for delivery of siRNAs.

Cargo	CPP/delivery system	proposed uptake mechanism	Reference
siRNA	MPG, MPGΔ^NLS^ (non-covalent)	non-endocytotic	[131]
siRNA	penetratin, transportan	n. d.	[104]
siRNA	Tat^47–57^, Tat-derived oligocarbamate	n. d.	[90]
siRNA	penetratin	n. d.	[230]
siRNA	H3K8b, H3K8b(+RGD)	endocytotic	[172]
siRNA	stearyl-R_8_	endocytotic	[232]
siRNA	Chol-R_9_	endocytotic	[121]
siRNA	MPGα	endocytotic	[236]
siRNA	R_8_-MEND (siRNA/stearyl-R_8_core)	endocytotic	[187]
siRNA	EB1, MPGΔ^NLS^, bPrPp	endocytotic	[76]
siRNA	Tat^48–60^, penetratin	endocytotic	[106]
siRNA	RVG peptide	n. d.	[234]
siRNA	rCPP	endocytotic	[181]
